# A microtomographic and histopathological evaluation of dental cements as late-stage peri-implant complication in a rat model

**DOI:** 10.1038/s41598-024-66353-x

**Published:** 2024-07-16

**Authors:** Bhuvana Lakkasetter Chandrashekar, Claudia C. Biguetti, Alexandra Arteaga, Andres J. Miramontes, Evelin Rios, Danieli C. Rodrigues

**Affiliations:** 1 Department of Bioengineering, University of Texas at Dallas (UTD), 800 W. Campbell Rd, Richardson, TX 75080 USA; 2 School of Podiatric Medicine, The University of Texas Rio Grande Valley, Harlingen, TX 78550 USA

**Keywords:** Pathogenesis, Risk factors, Signs and symptoms, Materials science

## Abstract

Cement mediated peri-implantitis accounts for 1.9–75% of dental implant failures associated with peri-implant diseases. This study evaluated the biological impact of dental cements on osseointegrated implants using Lewis rats. Twenty-two rats were distributed into 6 groups: negative control (NC) soft diet (SD), and hard diet (HD); positive control SD and HD (n = 3); Implant + bio-ceramic Cement (BC) SD and HD which included contralateral Sham sites (n = 5). Titanium implants were placed on either side of the maxillae and allowed to heal for 14 days. Later, both sides of experimental groups underwent a re-entry surgery to simulate clinical cementation. The right side received 0.60 mg of BC. At 14 days post cement application, maxillae were harvested for clinical, microtomographic, and histological evaluations. Clinical and microtomographic evaluations indicated evidence of extensive inflammation and circumferential bone resorption around BC implants in comparison to NC. Histology revealed cement particles surrounded by inflammatory infiltrate in the implant area accompanied by biofilm for SD groups. Both sides of BC indicated intensive bone resorption accompanied by signs of osteolysis when compared to NC. Cemented groups depicted significantly lower bone to implant contact when compared to NC. In conclusion, residual cement extravasation negatively impacted osseointegrated implants after re-entry surgeries.

## Introduction

Dental implants have been widely used as the structural and functional replacement for missing teeth and are highly successful leading to a global market size of $4.15 billion in 2022^1^. Fixed implant restorations can either by screwed or cemented on to the implant or by a combination of both techniques^2^. Cement retained restorations are preferred over screw retained systems due to various advantages such as desired loading characteristics, occlusal contact, simplicity, reduced costs and improved aesthetics^3–6^. However, residual/excess cement is a well-known risk factor for peri-implant disease and failures^7^. Despite literature highlighting the necessity of better practices to decrease the risk for extravasation of residual cement in peri-implant submucosa, it is a frequent clinical complication, and not all periodontists or oral surgeons receive equal training. In a survey conducted by American dental schools, it was demonstrated that cement loading procedures in crowns can vary drastically^8^. Additionally, for aesthetic reasons, clinicians may prefer to insert prosthetics at margins greater than 2 mm into the peri-implant sulcus. However, other studies have demonstrated difficulty in removing excess cement when margin restorations exceed 1.5 mm, and when the cement is finally removed, extensive scratching can occur on the abutment surface^9,10^.

Dental implant failures can further be classified into early and late implant failures based on the time of their occurrence after implant placement^11^. While early failures are mainly associated with inability to achieve osseointegration, late failures are affiliated to the loss of osseointegration^11,12^. Early implant failures are usually caused due to lack of primary stability, surgical trauma and infection, and late failures are mostly caused due to occlusal overloading and/or peri-implantitis^13^. Infectious conditions such as peri-implantitis could be attributed to the development of a biofilm on the implant surface due to various factors that include excessive cement particles, smoking, previous periodontal history and genetic factors^14,15^. Although peri-implantitis can be considered a late-stage complication, the early onset of such a condition has been associated with local risk factors such as excess dental cements^16,17^. Additionally, these residual dental cements can disturb tissue homeostasis, triggering chronic inflammatory response.

Peri-implant (PI) disease is defined as a plaque-associated pathological condition occurring in tissues surrounding osseointegrated dental implants. It is initiated by a destructive immune/inflammatory response in the peri‐implant connective tissue that gradually progresses to the marginal supporting bone^18,19^. The reversible inflammatory reaction restricted to soft peri-implant tissues can be categorized as peri-implant mucositis, while the progression to bone is classified as peri-implantitis^18^. Peri-implantitis is associated with a characteristic biofilm formation on the implant surface and submucosal excess cement deposits have been included among the parameters for Implant Disease Risk Assessment (IDRA)^20^, because of the high risk for biofilm development leading to infectious inflammatory conditions. The genesis for cement mediated peri-implantitis is associated with increased roughness of implant surfaces due to residual cements that can trap bacteria^21^. Additionally excess cements have also been considered as a predisposing factor for peri-implant tissue inflammation since they promote plaque accumulation due to retention of bacteria^21^. These observations suggest that biofilm formation on a rough cement surface in the submucosal region is essential to trigger a destructive inflammatory response at the peri-implant attachment site, since it is also the primary etiological factor for peri-implantitis. However, other studies have shown that an adverse foreign body reaction (FBR) to cement particles seems to be the primary triggering factor for early or late development of peri-implant diseases^22,23^. Thus, the predominant type of chronic inflammatory immune response leading to peri-implant disease associated with cements, infections or adverse FBR is still unclear. Also, these findings from the literature suggest distinct inflammatory mechanisms depending on cement compatibility accompanied by the presence or absence of biofilm/plaque accumulation.

Investigation of implants with clinical evidence of peri-implantitis showed that 81% of these implants were associated with excess luting agent^24^ indicating that retained cement could be a major contributor to peri-implant disease. Later, a systematic review showed that 33–100% of peri-implant diseases in cement-retained implant systems are associated with excess cement^25^. Previous studies investigating host cell and bacterial response to 4 commercially available dental cements: bioceramic (BC), zinc phosphate (ZP), resin-modified glass ionomer (RMGIC) and resin (R)^3^, depicted that pre-osteoblasts and macrophages were more sensitive to cements since they indicated lower cell proliferation rates. This suggests that exposure of host cells to different luting agents can disrupt tissue homeostasis at the peri-implant interface. Additionally, this study clearly indicated that bacteria could attack and alter cement morphology increasing surface roughness, which can trap microorganisms and promote biofilm development. Interestingly, host cells were sensitive to all cement compositions investigated with certain compositions (ZP, RMGIC, R) not meeting the minimum cytotoxicity criterion (ISO 10993-5). Additionally, it has also been demonstrated that different dental cement compositions promote corrosion of titanium surfaces when in contact^26^.

Previous studies illustrate the negative impact of dental implant extravasation on implant surface and peri-implant health^24,27^. However, the field lacks a comprehensive explanatory model for peri-implant diseases associated with residual luting agents. Consequently, most knowledge in the field is based on clinical observations and histopathological analysis from biopsies in case series or case reports^24^, making it difficult to develop personalized, innovative, and regenerative strategies to treat or prevent this biological complication based solely on empirical approaches used for the treatment of classic peri-implantitis or periodontitis. Therefore, by using a rat model of oral osseointegration and methodological tools combining MicroCT, histology, and immunohistochemistry, this study will allow answering the following research questions: Can residual dental cements lead to an adverse foreign body reaction (FBR) at the site of implantation? Is plaque accumulation a key/determinant factor driving peri-implant tissue breakdown associated with dental cements exposure, or mostly a synergistic cofactor to increase inflammation along with FBR triggered by excess dental cements? What is the extension of oral tissue breakdown in different scenarios of cement application post implant placement? By answering these questions, this histopathological study in a rat model aims to provide a better understanding of the effect of submucosal dental cement on adverse immune response leading to development of peri-implant diseases and on oral health in distinct clinical scenarios.

## Results

### Wound healing/scratching assays

**Figure 1:**
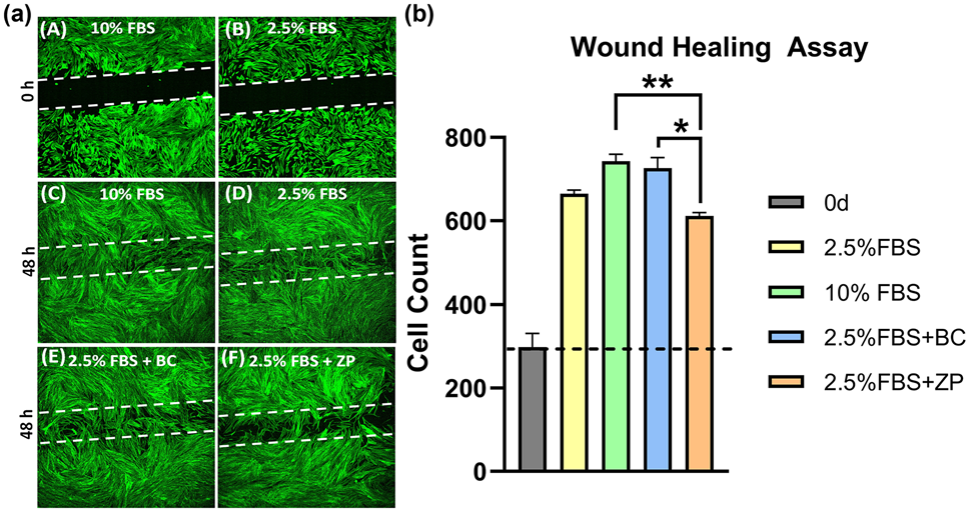
(**a**) Spinning disk microscopy images depicting wound introduction at 0 h (**A**,**B**), HGF migration after 48 h in media containing 10% and 2.5% FBS (**C**,**D**) and after 48 h exposure to media exposed to BC and ZP dental cements in media containing 2.5% FBS (**E**,**F**). While (**C**) and (**D**) indicate almost complete closure of the wound, depicting highest rate of HGF migration after 48 h when compared to 0 h, (**E**) indicates minimally reduced cell migration at the same time point. However, (**F**) indicates the least cell migration depicting the negative influence of ZP composition on wound healing and cell migration. White dotted lines represent the area of wound before and after cell migration. (**b**) Quantification of cellular counts within the scratches introduced during wound healing assay from spinning disk microscopy. * Indicates significant differences (p < 0.05) between groups (n = 4).

The scratching assay demonstrated that BC cement was more suitable for in vivo studies since they depicted higher cell counts in the scratch when compared to the ZP cement. BC cements were favorable for HGF proliferation that could correspond to biological seal formation in vivo (Fig. 1b). Additionally, qualitative data indicated that the scratch/wound introduced in the well plates were populated with a higher count of HGF for BC cements when compared to ZP (Fig. 1a). Moreover, BC cements also depicted the close cell count to control groups once again proving to be more cytocompatible than ZP as demonstrated by previous studies^3^. Therefore, BC cement was chosen for all in vivo work.

### Clinical evaluation

**Figure 2:**
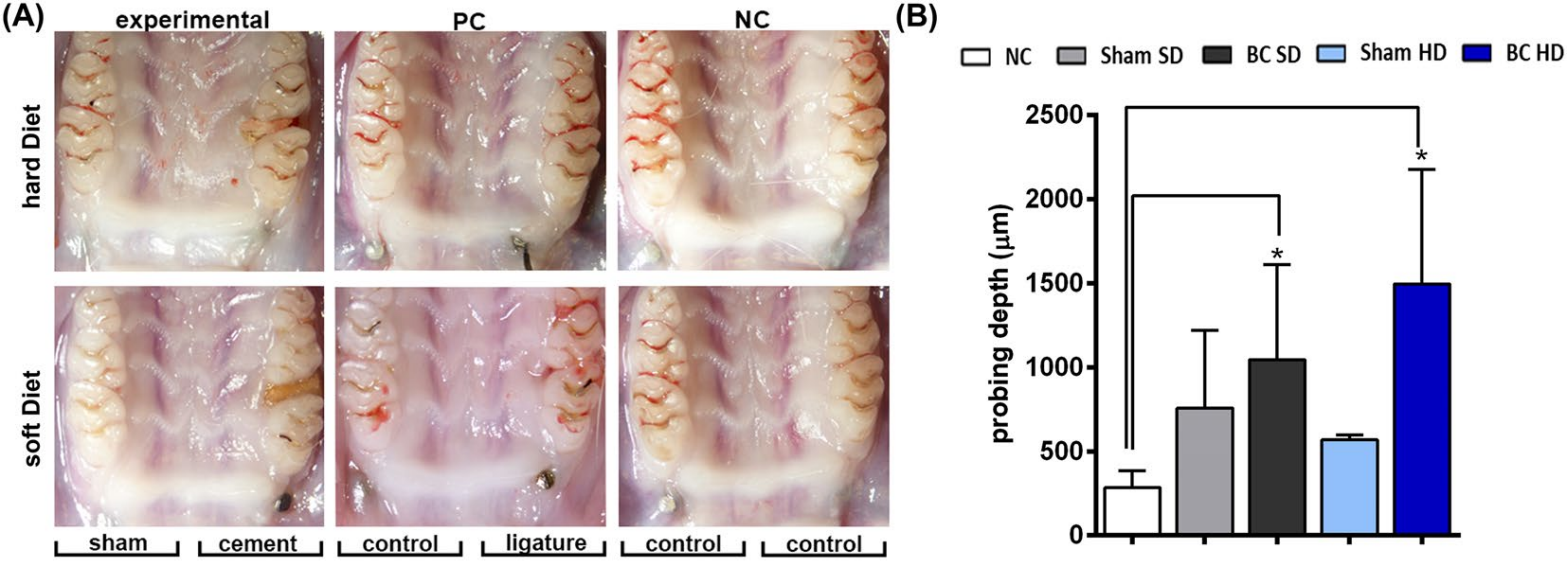
(**A**) Optical microscopy images indicating various clinical parameters evaluated: implant healed after 14 days of placement (dotted circles); plaque accumulation (red arrows) and shifting of adjacent teeth (blue dotted lines). (**B**) Probing depth measurements after late cement application at the adjacent periodontal teeth. *Indicates significant differences in probing depths when compared to negative controls.

Macroscopic evaluation for BC exhibited persistent implant head exposure, hyperemia (redness), hyperplasia (hyperplasia), and plaque accumulation at adjacent teeth of cemented sides for both SD and HD (Fig. 2A). On the other hand, NC and Sham groups presented healed peri-implant mucosa with no evidence of inflammation (Fig. 2A). Shifting of adjacent teeth at the site of cement application was observed for both SD and HD groups (Fig. 2A). NC and Sham SD groups also illustrated a degree of teeth separation, while no molar shifting was observed in control groups under HD (Fig. 2A). In addition to these parameters, the number of animals experiencing implant loss/mobility were also quantified to assess complete implant failure. While one rat belonging to the positive control hard diet group depicted implant loss/mobility, one more rat from the SD cemented group also indicated implant loss/mobility. Quantification of probing depths at adjacent periodontal tissues indicated significantly higher values for the cemented groups when compared to the negative controls (Fig. 2B).

**Figure 3:**
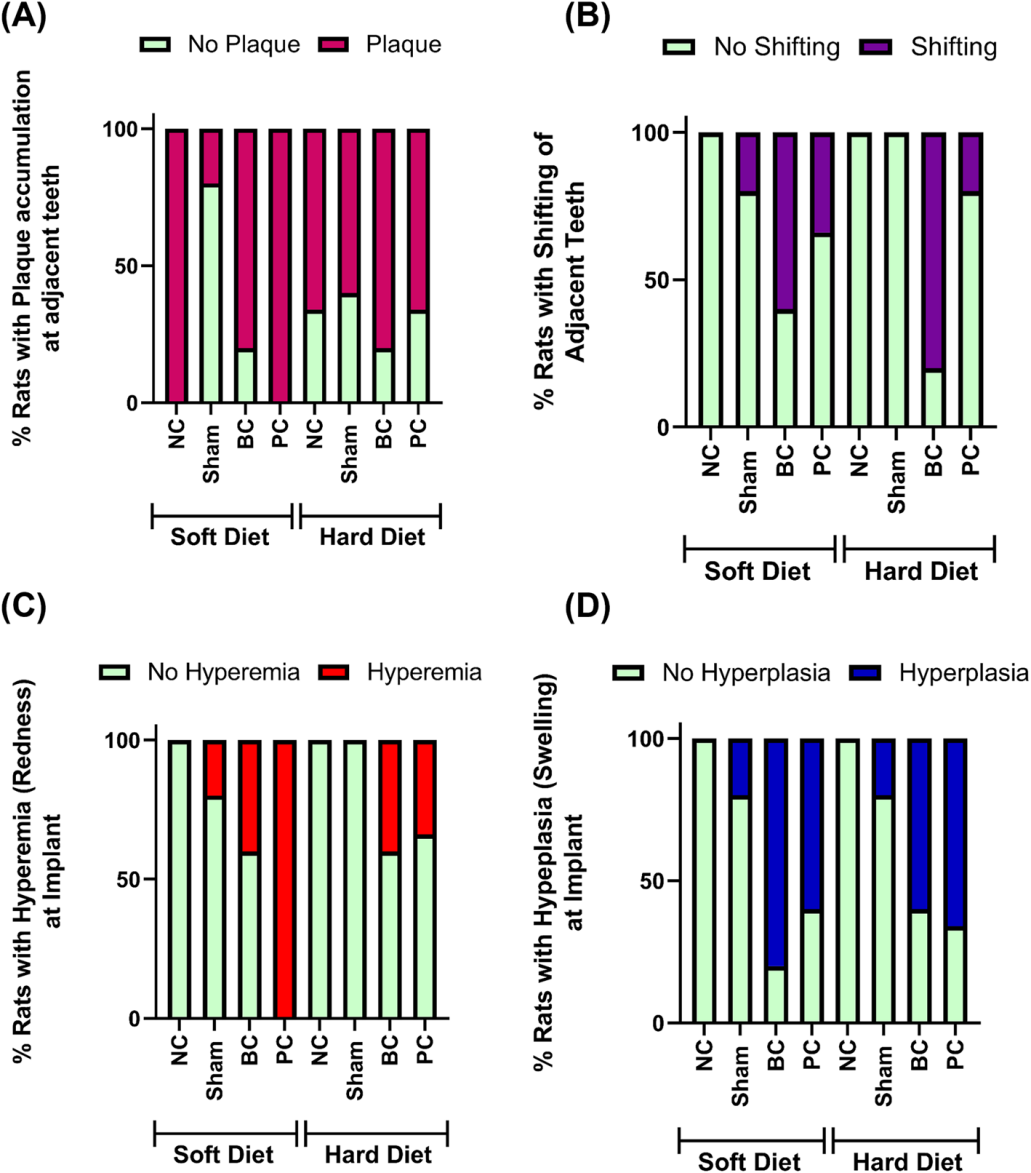
Quantification of % rats with plaque accumulation in the adjacent teeth (**A**), shifting of adjacent teeth (**B**), with hyperemia in the peri-implant region (**C**) and with hyperplasia in the peri-implant region (**D**) using chi-square analysis.

Results from chi-square analysis depicted a statistically significant relationship between samples with and without plaque accumulation at adjacent teeth (Fig. 3A), molar shifting (Fig. 3B), hyperemia (Fig. 3C) and hyperplasia (Fig. 3D) at implant site. As expected, the SD favored plaque accumulation in all groups while cemented under both SD and HD groups exhibited higher molar shifting in comparison to the controls and Shams. Similarly, a higher percentage of rats depicted hyperplasia for cemented groups in comparison to the control.

### MicroCT analysis

**Figure 4:**
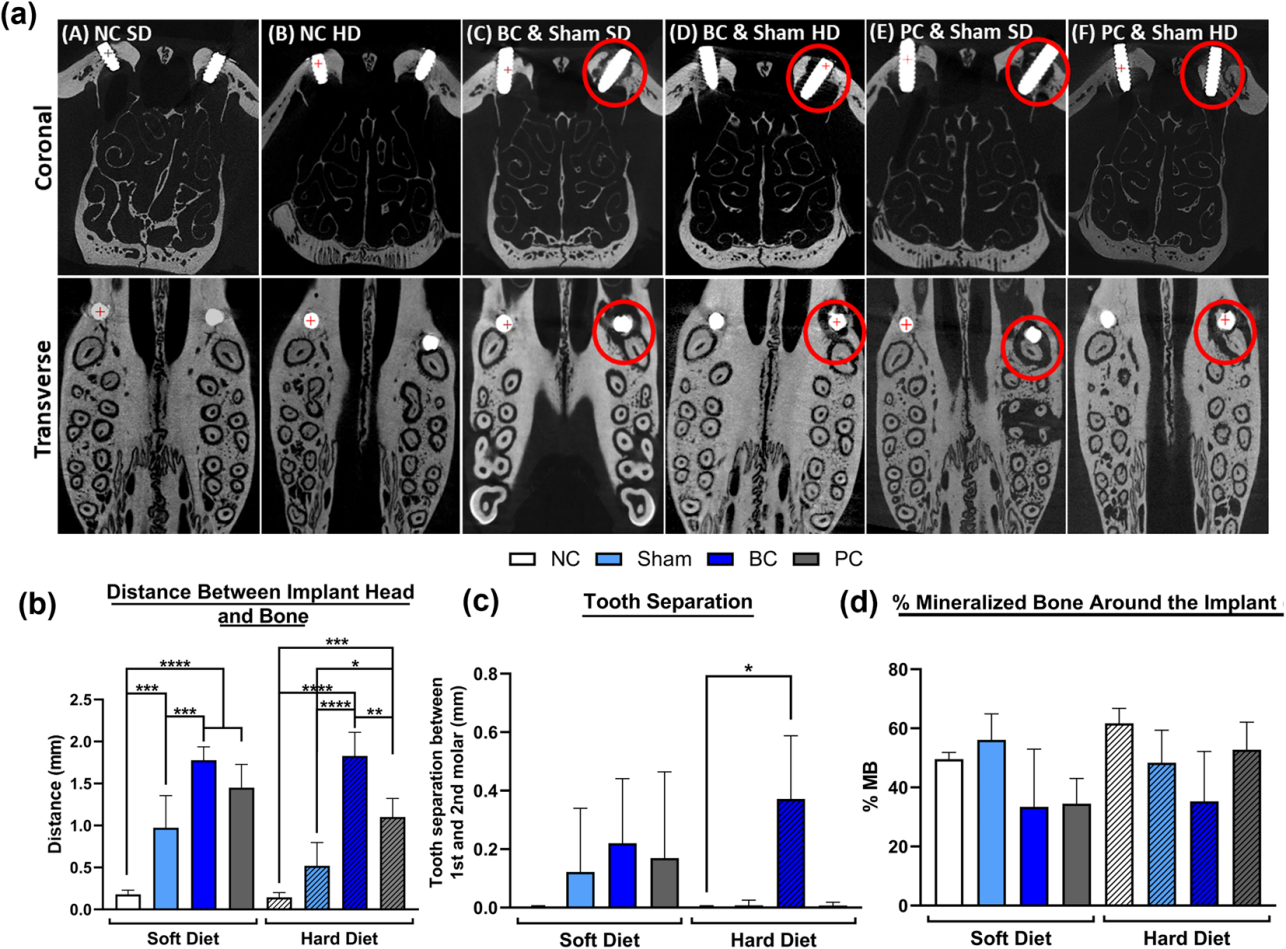
Microtomographic images of rat maxillae containing implants in the coronal and transverse view (**a**) depicting osseointegration on both sides at day 28 post-implant placement (**A**,**B**); Maxillae after 14 days of reentry surgery (left side) and cement application (BC, right side) (**C**–**F**); Distance between implant head and one around the implant (**b**) between all the groups; Tooth separation between 1st and 2nd molars (**c**); and Percentage of mineralized bone around the implants (**d**). Red circles indicate severe bone loss/resorption in the peri-implant region. * Indicates significant differences between groups.

Negative controls achieved satisfactory bone formation with no signs of bone resorption or loss of bone around the implant (Fig. 4a). Contrarily, cemented samples demonstrated circumferential and extensive bone resorption around cemented implants in comparison to the NC and Sham groups, under both HD and SD consistencies (Fig. 4a). Quantification of the distance between the implant head and bone crest depicted those implants with cement application had significantly higher values in comparison to non-treated implants (controls or Shams), irrespective of the type of diet (Fig. 4b). Additionally, the Sham sites under SD consistencies also indicated significantly higher distances in comparison to the NC (Fig. 4b) and the Sham sites for both SD and HD consistencies depicted significantly lower distances in comparison to their respective cemented groups. Although, the PC samples had significantly higher distances than that of NC under both SS and HD, the cemented groups for HD had significantly higher distance between implant head and bone when compared to the PC. As an evaluation of the secondary effects of cements on adjacent periodontal tissues, quantification of tooth separation between the first and second molars depicted a significantly higher values between the cemented and NC groups for HD (Fig. 4c). Furthermore, for SD groups all the groups depicted an increased trend in the tooth separation when compared to the respective NC. Morphometric analysis revealed similar trends with BC groups exhibiting the least %MB surrounding the implant among all groups for both S and HD conditions, further confirming the effect of dental cements (Fig. 4d).

### Histopathological and histomorphometric evaluation

#### Standard H&E staining

##### Hard tissue/bone

**Figure 5:**
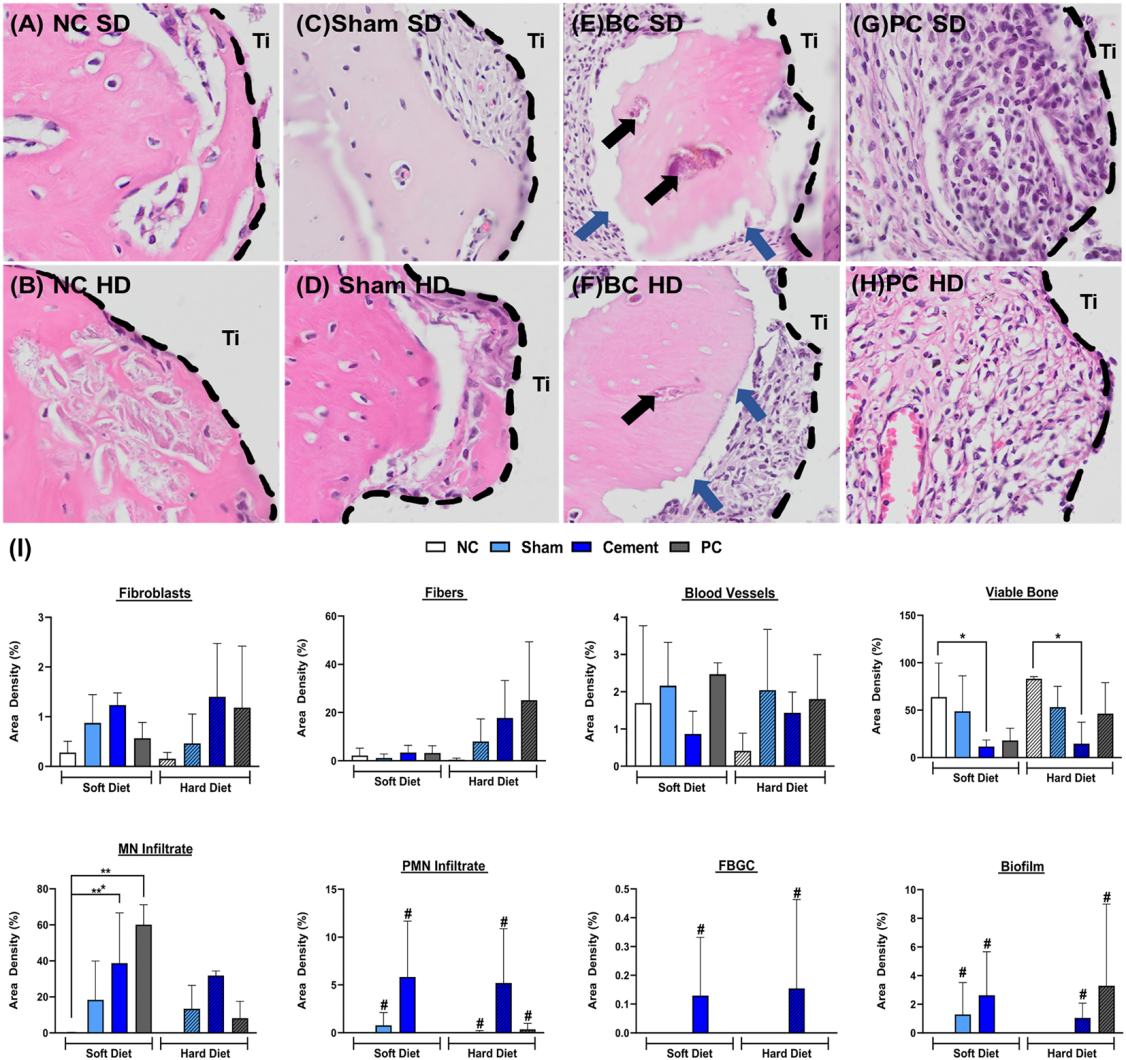
Histology representing peri-implant bone healing of negative control samples (**A**,**B**), delayed healing and secondary inflammation at Sham sites (**C**,**D**) and peri-implant inflammation, bone resorption in the cemented rats (**E**,**F**) and positive controls (**G**,**H**) maintained under soft and hard diet at 28 days after implantation, re-entry surgeries, cementation, and ligature placement, respectively. The panel displays an overview of peri-implant hard tissue; Ti stands for the implant area. Blue arrows indicate regions of bone resorption, inflammatory infiltrate and black arrows indicate biofilm trapped within the bone. Histomorphometric analysis of peri-implant hard tissue healing/inflammatory parameters for different groups (**I**). * Indicates significant differences between groups (p < 0.05). # Indicates the presence of the respective parameters unlike their negative controls.

At 28 days after implantation in the NC groups, the hard tissue was dominated by mature bone matrix encompassing osteocytes and vessels that appeared to be mineralized for both HD and SD (Fig. 5A,B). Similar histological dynamics of osseointegration were observed at the Sham sites that were subjected to reentry surgeries but didn’t receive cement. Nonetheless, they also appeared to demonstrate evident mature bone and collagen fibers when compared to the NC (Fig. 5C,D). Contrarily, BC and PC groups either depicted necrotic/non-viable bone with empty lacunae (depicted by red arrows) or no bone accompanied with biofilm entrapped (depicted by black arrows) in the bone matrix and bone structure depicted higher osteoclastic activity (Fig. 5E–H). Additionally, the necrotic bone was also accompanied by the presence of MN (mononuclear infiltrate), PMN (polymorphonuclear infiltrate) and FBGC (foreign body giant cells). Quantitatively, the NC under both SD and HD depicted significantly higher amount of intramembranous bone healing in the form of mature viable bone when compared to the BC groups (Fig. 5I). The experimental groups also depicted the highest number of fibroblasts among all the groups for SD and HD (Fig. 5I). However, an increase in fibrous connective tissue was only observed for HD consistencies in the experimental groups when compared to NC (Fig. 5I). Similar trend was observed for the PC samples (Fig. 5I). The non-viable bone was accompanied by significantly higher influx of MN infiltrate in the Sham, BC and PC groups under SD in comparison to the NC rats with comparable trend under HD (Fig. 5I). Additionally, the MN infiltration was accompanied by the presence of PMN infiltration, FBR with the presence of FBGC and biofilm (Fig. 5I).

##### Soft tissue

**Figure 6:**
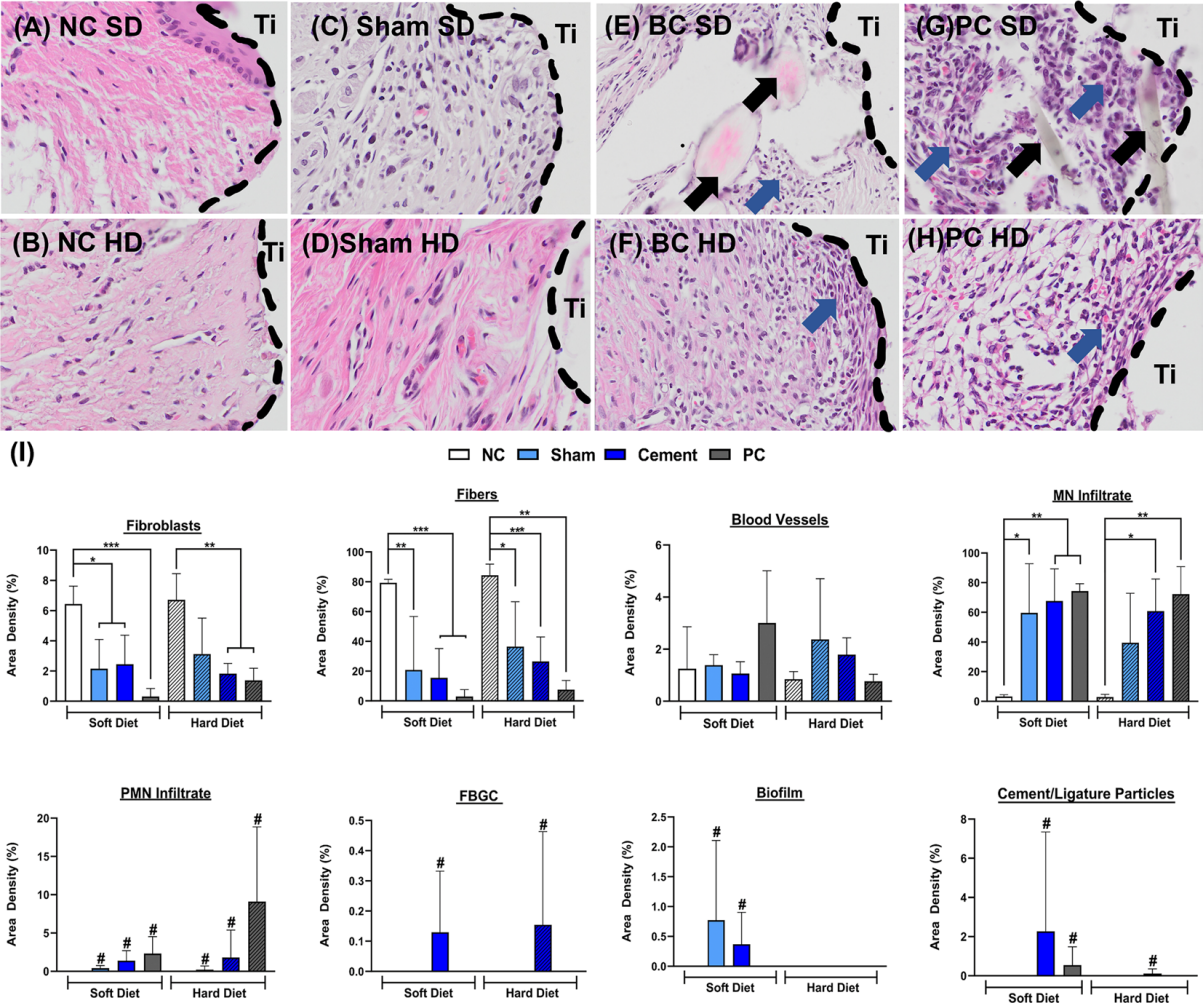
Histology representing peri-implant soft tissue healing of negative control samples (**A**,**B**), delayed healing and secondary inflammation at Sham sites (**C**,**D**) and peri-implant inflammation, round eosinophilic foreign bodies (cements) in the cemented rats (**E**,**F**) and positive controls with remnant ligature pieces (**G**,**H**) maintained under soft and hard diet at 28 days after implantation, re-entry surgeries, cementation and ligature placement, respectively. Panel displays an overview of peri-implant soft tissue; Ti stands for the implant area. Blue arrows indicate regions of inflammatory infiltrate. Black arrows indicate the presence of eosinophilic foreign particles (residual cements) or ligature fragments. Histomorphometry of peri-implant soft tissue healing parameters for different groups (**I**). * Indicates significant differences between groups (p < 0.05). # indicates the presence of the respective parameters unlike their negative controls.

Qualitative histopathology of the soft tissues was similar to that of the hard tissue in the NC rats (Fig. 6A,B) and the presence of chronic inflammatory infiltrate (Fig. 6E–H) in the BC and PC groups. Interestingly, only the BC rats under SD indicated the presence of round eosinophilic foreign particles which we hypothesize to be the residual cements particles (depicted by black arrows) since they were accompanied by the presence of biofilm and adverse foreign body reaction in the form of PMN and FBGC (depicted by red arrows). Nonetheless, the BC rats under HD also indicated signs of chronic inflammation by the presence of PMN and FBGC. The same was true for the PC rats, except that soft tissue sections for SD depicted the presence of ligature particles instead of cement particles. Although the Sham groups do not compare with the BC and PC groups, they depicted irregular structure and increased inflammatory infiltrate in comparison to the NC rats (Fig. 6C,D). Histomorphometry of the soft tissue corroborates with the qualitative analysis depicting significantly lower amounts of fibroblasts and fibers in the cemented and PC groups including Shams under both the diets indicating insufficient biological seal development (Fig. 6I). Although no significant differences were observed for the quantity of blood vessels observed, all the groups confirmed inflammation through significantly higher MN infiltrate with the presence of PMN and FBGC in comparison to the NC (Fig. 6I). Soft diet also favored biofilm formation in the soft tissues for the cemented rats (Sham and BC) as quantified.

#### Goldner’s trichrome (GT) staining

**Figure 7:**
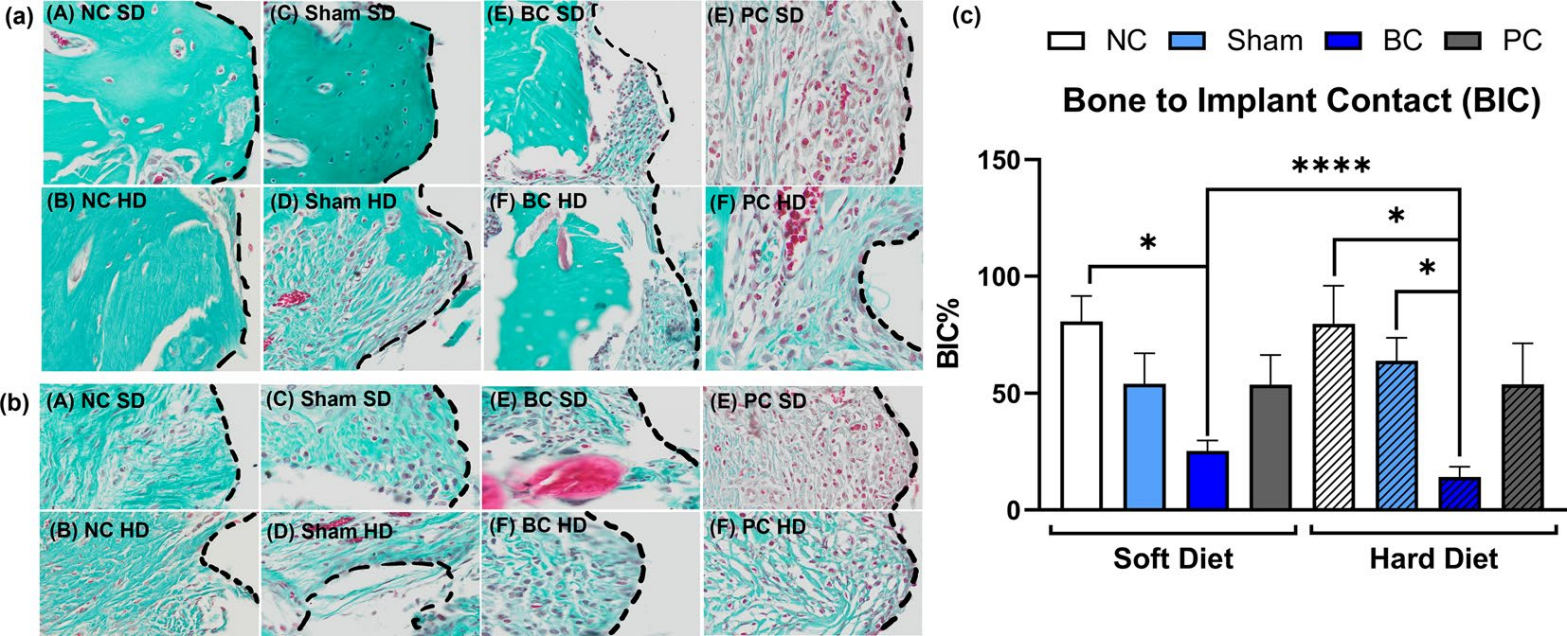
Histology representing peri-implant tissue (**a**,**b**) healing in GT stains for negative control samples (**A**,**B**), delayed healing and secondary inflammation at Sham sites (**C**,**D**) and peri-implant inflammation, round eosinophilic foreign bodies (cements) in the cemented rats (**E**,**F**) and positive controls (**G**,**H**) maintained under soft and hard diet at 28 days after implantation, re-entry surgeries, cementation and ligature placement, respectively; BIC quantification indicating mean ± SD for BIC% of bone formation/loss in the peri-implant area (**c**). * Indicates significant differences between groups (p < 0.05).

While BC groups indicated severe bone loss, necrosis, and inflammatory infiltrate, qualitatively, Sham groups depicted immature bone matrix as compared to the negative controls (Fig. 7a,b). Following histological analysis of BIC%, it was confirmed that for both SD and HD consistencies, the cemented groups (BC) had significantly lower BIC% in comparison to their negative controls (Fig. 7c). BC groups had significantly lower bone formation when compared to the Sham under HD condition (Fig. 7c). Although this didn’t hold true for the SD group, the same trend was observed for both diets with BC groups depicting the least amount of BIC%. Interestingly, amongst all the analysis so far, the BIC% was significantly lower for the HD group than the SD group amongst their respective BC samples (Fig. 7c).

## Discussion

The presence of foreign materials like excess cement in the adjacent peri-implant tissues have also been identified as contributing factors to peri-implant pathology. Therefore, it is clear from previous studies that PI is a multifactorial disease and various local risk factors including, but not limited to inadequate plaque control, history of periodontitis and presence of excess cements^28^. However, it is important to know that one or more factors in combination with each other can contribute to PI disease progression. Unlike periodontitis, which is attributed to generalized loss of hard tissue support, progression of peri-implantitis is conditioned by factors predisposing biofilm accumulation that under susceptible conditions, initiate complex inflammatory response^29^. Excessive plaque accumulation has already been identified as a predisposing factor for inflammation associated with peri-implantitis, mainly when accompanied by other determinant features. As of plaque-associated peri-implantitis, various local contributors such as surgical and prosthetic features, soft and hard tissue characteristics have been identified as precursors to biofilm formation around dental implants, thereby leading to inflammatory conditions^29^. Importantly, previous studies have emphasized residual dental cements as a major precipitant or triggering factor for peri-implantitis, further establishing that change in surface topography/roughness as with of dental cements can further accelerate the process. Therefore, all these studies further accentuate the need for more mechanistic understanding of peri-implantitis as a multifactorial disease to target multiple factors causing it.

Often dental implant restoration systems involve a cementation step that could lead to variable amounts of cement retention at the junction of the prosthetic crown and the surrounding host tissues^30^. Application of the right amount of cement inside the crown is mostly based on the clinician’s expertise to prevent residual cements from being profligate deeper into peri-implant spaces, which is unmistakably challenging to be addressed by manual instruments or oral hygiene^30^. Besides, reduction of cement related complications is also greatly dependent on the design quality and expertise involved in prosthesis placement. Verily, cement-retained restorations accompanied by marginal misfit have shown to be inducing early crestal bone loss in comparison to the accurately fitted implants over a mean period of 3 years post-implantation^31^. Moreover, it is not contingent that residual dental cements can foster an appropriate environment for biofilm accumulation. Despite literature highlighting the possible antibacterial activity of dental cements^32,33^, it has also been shown that such activity is time-dependent and that under extended time periods, excess cements can serve as crevices for bacterial agglomeration initiating local infection^27^. Additionally, it has been widely reported that as a result of physiological variability amidst different patients, various bacteria present in the oral environment can easily contaminate dental cements^27^. On the contrary to the claim that dental cements possess anti-bacterial properties, previous in vitro evaluation of various dental cement compositions demonstrated that they didn’t necessarily contain any anti-bacterial agents^3^. In addition, dental cements may differ in their compatibility^3^. Since there is a variety of options for cementation, we first determined the most biocompatible to HGF in a scratching assay. For this assay, we compared ZP, a gold standard^34^ for implant prosthesis retention, and BC, which has been shown good cytocompatibility in our previous work^3^. As BC confirmed higher compatibility than ZP, it was utilized for all the following in vivo studies.

Osseointegration can be viewed as a favorable FBR in balance with the host^35^. With this consideration, bone loss in peri-implant area could be correlated with a loss of this immune-driven balance^35^. Although the precise mechanism of pathogenesis behind such a bone loss is not clearly understood, there are hypothetical models that suggests cement remnants and Ti particles as factors influencing the breakdown of the established balance between host tissue and the implant^36^. This can serve as a triggering factor to activate macrophages, upregulating the osteoclastic activity, thereby inducing bone resorption^36^. It is well known that the disruption of osseointegration relies upon the balance between the immune system and the trilogy of patient characteristics, clinician’s surgical expertise and the design of the implant^36^. However, these factors are trivial at the early stages of implant healing. Nonetheless, in case of marginal bone loss occurring at later stages of implantation, several other reasons have been recognized as triggering factors for adverse FBR and bone loss^35^. This has been previously referred to as “late disbalance of foreign body equilibrium”^37^. Factors such as occlusal over loading, cement remnants and bacteria have been identified as leading causes of such type of failures^37^.

Understanding the mechanisms associated with excess cements may contribute to the improvement of biomaterials and/or prevention of cement mediated implant failures. In the current study, a rat model was used to mimic the late cement application in healed dental implants using an oral osseointegration model that has been previously established^38–40^. By utilizing clinical, microtomographic and histological analysis, this study was able to characterize cement mediated late implant failures in comparison to successful oral osseointegration of Ti implants and ligature induced peri-implantitis.

Macroscopic clinical evaluation depicted suitable peri-implant mucosa healing in the negative control groups while other signs of inflammation such as redness, swelling and plaque accumulation at the implant site and adjacent teeth were observed for the experimental groups in addition to implant disintegration (Fig. 2A). This confirms compromised host tissue response and implant surface integrity. This is in conjunction with the previous in vitro studies that evaluated the impact of dental cements on implant corrosion behavior^26^. Lack of favorable implant surface characteristics can further lead to production of Ti ions and particles that can exacerbate the immune response in peri-implant tissues^41^. Further, soft tissue around the implant with the various aforementioned clinical symptoms can mostly be associated with peri-implantitis. It has been established that the quality and quantity of peri-implant soft tissues is a crucial factor for dental implant success^42^ while the results from clinical evaluations point to the detrimental impact of submucosal dental cements on soft tissue health around the implant. Moreover, measurement of the probing depth at the periodontal tissues was used to define the extent or spread of cement mediated peri-implantitis (Fig. 2B). Evidently, cemented groups had higher probing depths at the first molars for both soft and hard diet consistencies indicating development of inflammation and infection in the periodontal tissues. Additionally, this was accompanied by extensive plaque accumulation leading to shifting of the adjacent teeth indicating vulnerable periodontal bone quality. All in all, the results from the clinical evaluation depicted the onset of peri-implantitis and secondary periodontal inflammation.

Qualitative microtomographic analysis of the peri-implant region in various groups depicted consistent and satisfactory ossification for the NC samples while the BC and PC groups showed loss of supporting bone depicting disruption/loss of osseointegration that was probably achieved before the introduction of dental cements. This was further confirmed by the higher distance between implant head and bone crest for the BC, PC and Sham sites when compared to negative controls. This corresponds to a significant compromise of peri-implant bone health in both the peri-implant and periodontal regions. Moreover, assessing the extent of teeth separation between first and second molars further confirmed the induction of secondary bone loss. Further, evaluation of the %MB around the implants corroborated with these results indicating threatened bone health when using dental cements.

Favorable FBR in PI region is regarded as a nonspecific immune-driven reaction comprising of complement and macrophages as major elements of the subsequent immune-inflammatory balance leading to implant integration^43,44^. On the other hand, when this process of healing is disrupted by various other factors, it could lead to prolonged persistence of inflammatory phase commonly termed as chronic inflammation^45^. This in case of dental implants has been commonly related to the pathogenic condition of peri-implantitis which in addition to chronic inflammation is also associated with microbial challenge^46^. Although PI disease is often a biofilm-mediated infectious process, marginal bone loss could also be due to other immunological reasons^37,44,47,48^. Correspondingly, a disbalance in the stable immune system upon achievement of osseointegration in an implant system has the potential for implant rejection^48^. Given what has been established in this regard, elements such as surface wear, type of implant material, or contaminated particles could enhance the resulting immunological responses^49^.

To investigate the mechanisms underlying impaired osseointegration due to residual dental cements, different types of histomorphometry analysis were performed, comparing unsuccessful and successful osseointegration sites. Presence of necrotic bone and biofilm entrapment at the experimental sites unlike the NC groups and increased fibrous connective tissue in BC group instead of the presence of mineralized bone post 28 days of implantation is not only an indication of failed osseointegration, but also fibrous encapsulation of the implant indicating implant failure^50^. Additionally, as observed in the PC groups, presence of similar features confirmed induction of PI diseases enabling us to confirm that residual dental cements could have caused a similar effect. Intriguingly, increased fibrous tissue and decreased BIC% in the HD groups indicate that the type of diet could influence PI disease progression under susceptible conditions. Although it was hypothesized that SD could be more influential for PI disease development, under the presence of foreign particles like dental cements and Ti ions, HD could impart the same effect in fact with a higher probability. This could be because the HD could reflect in higher food retainment on the teeth than SD under the same oral environment and salivary influx. Moreover, HD could result in greater mechanical loading on the implant and in the absence of satisfactory osseointegration (because of cement remnants) could be conducive to implant failure. However, there is much to explore to understand the effect of the type of diet on implant integration. Furthermore, loss of osseointegration and increased inflammatory influx at sham sites could be an indication of secondary inflammation and infection, accompanied by delayed healing. Although, these areas were not directly exposed to dental cements, indirect contact could also trigger an inflammatory response and negatively impact the healing process.

This study confirmed that the extended presence of foreign particles like dental cement remnants can adversely alter the Ti-tissue interface. Additionally, dental cements can also negatively affect the corrosion behavior of Ti implants, in turn contributing to a synergistic inflammatory response along with the dental cements to release toxic Ti ions and particles, further altering the biocompatibility of Ti-based implants. Comprehensively, presence of submucosal dental cements induces exacerbated chronic inflammation in both soft and connective tissues, allied with an extension of fibrosis and proinflammatory phase resulting in hostile healing and osseointegration. Clinically, the findings from this study serve as a motivation for the development of targeted therapies for cement-mediated dental implant failures. This study also places emphasis on the need for standardizing cementation procedures and protocols to have no residual cements. Further, the methods utilized for the cement removal from implant surfaces also need to be personalized for specific patient needs and clinical scenarios. Finally, it is important to consider that the current study illustrates a model to reproduce inimical host response in response to the presence of unfavorable foreign bodies such as dental cements.

As with any in vivo study, current study has its own limitations. Inclusion of separate groups of animals for sham surgeries without the introduction of cements would better explicate the independent role of dental cements in the resulting pathogenic environment. Future molecular studies are required to better understand the markers and mechanisms involved in such challenging in vivo conditions. Therefore, within the limitations of the current study, cement particles could be viewed as foreign material that are a budding source of adverse FBR leading to severe loss of supporting alveolar bone around dental implants. The results point out that submucosal residual cements induced exacerbated inflammatory response in both soft and hard tissue in the peri-implant region.

## Methods

### Cements and cement selection

Selection of cements for in vivo experiments was based on in vitro experiments with two commercially available dental cements^3^. Wound healing experiments were conducted with a zinc phosphate (ZP) cement (Fleck’s Mizzy, Cherry Hill, NJ) and a permanent biocermaic (BC) dental cement (Ceramir Bioceramic Implant cement, Doxa Dental Inc., Uppasala, Sweden). Cement selection for in vivo experiments were based on the results from the in vitro assays.

### Wound healing/scratching in vitro assays

Human Gingival Fibroblasts (HGF) were plated on to a 6-well plate with a seeding density of 60,000 cells per well and allowed to proliferate up to 70% confluency to ensure a monolayer of cells. BC and ZP dental cement disks were prepared according to the manufacturer’s instructions and incubated with 1-ml of cell culture medium in a separate 24-well plate for 24 h in triplicates. The BC and ZP dental cement groups were considered as the conditioned media and were utilized as the experimental group. At 70% confluency, a scratch was introduced at the center of the wells with a 100 µl pipette tip in each well of the 6-well plate, followed by incubation with 10% fetal bovine serum (FBS) medium and 2.5% FBS medium as control groups. The cells were then allowed to migrate until the scratch in the 10% FBS group was completely covered. Samples were divided into six groups: Wells at 0 h after the scratch introduction, 2.5% FBS 48 h, 10% FBS 48 h, 2.5% FBS + BC 48 h, and 2.5% FBS + ZP 48 h. The cells were later fixed and stained with 0.3 µM of 4′,6-diamidino-2-phenylindole (DAPI, Invitrogen) and Alexa Fluor 488 dye (Invitrogen). Samples in wells were maintained in 1.5 ml of PBS prior to fluorescent imaging using a spinning disk confocal microscope (Olympus SD-OSR) at 10× magnification. The number of cells that migrated into the scratch were quantified by QuPath^51^ (Version 0.4.3) software from the six groups and were statistically analyzed for significance (n = 4) and the results were presented as mean ± standard deviation.

### Surgeries: implant placement, maxillary implantation, and cement application

A total of 19 10–12-week adult male Lewis rats (Charles River Laboratories, Wilmington, MA, USA) weighing approximately 250–350 g, were distributed into 6 groups: negative controls (NC) soft (S) diet and hard (H) diet after 28 days (n = 3 yielding in 6 NC samples per group); positive controls (PC) with ligature (n = 3); Implant + Cement (BC) S and H (n = 5). The animal strain and number of animals were determined based on previously published similar studies^38,39^. A detailed description of the groups and time points is given in Table 1.

**Table 1:** Distribution of Lewis rats into different experimental and control groups based on the types of diet and time points.

Diet	Group	Nomenclature	Experiment	Euthanasia
Soft (SD)	Negative Control	NC SD	Only implant	At 28 days post implantation
	Positive Control	PC SD	Implant + Ligature	At 28 days post implantation/14 days post ligature
	Implant + Cement	BC SD	Implant + Cementation	At 28 days post implantation/14 days post cementation
	Sham	Sham SD	Implant + Re-entry surgery	At 28 days post implantation/14 days post re-entry surgery
Hard (HD)	Negative Control	NC HD	Only implant	At 28 days post implantation
	Positive Control	PC HD	Implant + Ligature	At 28 days post implantation/14 days post ligature
	Implant + Cement	BC HD	Implant + Cementation	At 28 days post implantation/14 days post cementation
	Sham	Sham HD	Implant + Re-entry surgery	At 28 days post implantation/14 days post re-entry surgery

The dental implants used in rats for this study were Grade 2 commercially pure titanium (cpTi) threaded pins (0.76 mm × 2 mm, Fairfax Dental Inc., Miami, FL, USA), as previously described^38^. All the implants were cut to be 2 mm in length using orthodontic pliers and were sequentially cleaned by sonication with acetone, de-ionized water, and ethanol for 15 min each. The implants were then dried at 60 °C in an oven after which they were sterilized using an autoclave. BC cement was prepared according to manufacturers’ instructions and loaded into an insulin syringe for cement application. The quantity of cement applied was quantified and standardized to be 0.60 mg by mimicking cement preparation and application using a weighing scale (data not shown). Rats were weighed before surgery and sacrifice to monitor weight. The animals were anesthetized by 4% isoflurane inhalation followed by intramuscular injection of 50–100 mg/kg of ketamine hydrochloride and 20–50 mg/kg of xylazine hydrochloride. Next, the anesthetized rats were placed in a dorsal decubitis position on the surgical table for surgery in the edentulous maxillary region. After positioning the rats, the surgical site received a lidocaine injection of 20 mg/kg with 1:100,000 epinephrine (Quala Dental Products, Nashville, TN, USA) for local analgesia and hemostasis. Surgical implantation was initiated by a 2 mm mucosal incision positioned 1 mm in front of the maxillary first molar to expose bone. This was followed by drilling a 0.67 mm implant bed with a surgical micromotor at 800 RPM (NSK Surgic Pro) with subsequent placement of a titanium screw (0.76 mm ⌀ by 2 mm) in the edentulous alveolar crest osteotome site using microneedle holders as per previously established osseointegration models^38,39^. This surgical implantation was performed on both the sides of the maxillae and allowed to heal with standard hard diet. Further 14 days after implant placement, a re-entry surgery was performed to expose the implant head while only the right side of the rat maxillae received the BC dental cement and left side was used as a re-entry surgery sham for the Implant + Cement groups. At this stage standard chow moistened with water was introduced in soft diet groups. Furthermore, the same procedure was repeated with a 6-0 silk ligature placement around the implant head for another set of rats that served as positive controls. The ligature was placed 14 days post-implantation accompanied with soft diet for plaque accumulation to induce PI disease. Additional negative control surgeries were performed on rats and sacrificed at 28 days after implant placement without cement application. Feeding, drinking, and grooming were monitored daily during the post-operative period. Animals were sacrificed by an overdose of pentobarbital sodium (Euthanasia III Med-Pharmex Inc., Pomana, CA, USA) at 14 days after cement application (28 days after implant placement).

### Clinical parameters evaluation

After sacrifice, images of the maxillae were captured using a stereomicroscope (Olympus, SMZ45T with SD-Fi2-L3 Camera, Shinjuku City, Tokyo, Japan) to track clinical features of healing/lack of healing and inflammation such as plaque accumulation at the implant site and adjacent teeth, exposed implant, hyperemia (redness), residual cements, hyperplasia (swelling), shifting of adjacent teeth, implant loss/mobility, implant discoloration and tooth mobility. The percentage of rats exhibiting these parameters were quantified and compared among various groups to understand the impact of cementation using chi-square analysis. Later, the extracted maxillae were placed in 10% neutral buffered formalin (NBF) for future microtomography and histology analysis.

### Microtomographic analysis (MicroCT)

Microtomographic analysis was used for the evaluation of implant position in relation to the surrounding bone and osseointegration around the implants. Following fixation in NBF for 48 h, samples were continuously washed for 24 h in water and placed in 70% ethanol for future processing. The whole maxillae containing implants were assessed by ultra-high-resolution MicroCT imaging (OT/CT, Milabs, Utrecht, Netherlands). Image acquisition was conducted at 50 kV, with a current of 0.21 mA and exposure time of 75 ms. Projection files were reconstructed by a vendor software and then converted to DICOM (Digital Imaging and Communication in Medicine) files using PMOD analysis software (PMOD Technologies LLC, Zurich, Switzerland) at voxel size of 20 µm. A software called ImageJ (Version 1.51, National Institute of Health, Bethesda, Maryland, USA) was utilized to quantify the percentage of mineralized bone and percentage of bone loss around the implants. The obtained MicroCT images were converted to 8-bit images before adjusting the contrast and brightness for better visibility. The exact images slices were separated from the original stack to create a sub stack of 10 image slices consisting of the peri-implant region. A constant region of interest (ROI) with a width and height of 150 × 150 pixels was chosen in the peri-implant bone region in each slice. A freehand selection tool allowed precise quantification of the area of bone around the implant. This was repeated for all the images in the sub stack and was averaged to obtain the percentage of mineralized bone (%MB) excluding the implant area. In Addition to this, the distance between the implant head and bone, and tooth separation between the first and second molars (in mm) was quantified for all the maxillae using Imalytics Preclinical (Gremse-IT GmbH, Aachen, Germany). This involved measuring the distance between the implant head and closest visible bone crest on the mesial, distal, buccal, and palatal sides of the implant. This measurement corresponds to the extent of bone loss around the implant, that could further be attributed as one of the important features of peri-implant diseases.

### Histological processing and staining

Post MicroCT scanning, rat maxillae were decalcified by placing in 10% ethylenediaminetetraacetic acid (EDTA)-2Na (Sigma, St. Louis, MO, USA) with two EDTA changes per week. After decalcification, the whole maxillae were reduced to transverse sections containing the implants. These samples were then processed in a tissue processor (Leica ASP300) for 12 h. The Ti implants were then carefully unscrewed from the maxillae using microneedle holders and embedded in paraffin blocks^39^. Six sets of 4 serial sections with a thickness of 5 µm were obtained from each rat. The progression of healing/inflammation was evaluated by staining the samples with hematoxylin and eosin (H&E) and Goldner Trichrome (GT) stain as per previous protocols^52^.

H&E-stained sections were used for the evaluation of soft and hard tissue integrity at the peri-implant interface. Evaluation of the soft tissue integrity at the mucosa-implant interface was conducted using the area from the alveolar bone crest to the point of implant emergence through oral epithelium of the peri-implant mucosa. On the other hand, hard tissue integrity was evaluated around implant threads in contact/adjacent to the alveolar bone, excluding the implant region that penetrated though the maxillary sinus. Histomorphometry at the soft tissue was conducted to quantify bloods clots, blood vessels, inflammatory cells, foreign body giant cells (FBGC), fibroblasts, fibers, epithelium, features of biofilm and any visible cement remnant particles in four histological fields, which were later averaged. Hard tissue histomorphometry was utilized to quantify blood clots, blood vessels, inflammatory cells, FBGC, fibroblasts, fibers, osteoblasts, viable and non-viable bone, and any entrapped biofilm in six histological fields per section, which were averaged.

GT-stained sections were used for the evaluation of bone to implant contact percentage (BIC %) as previously described^39,41^. In short, BIC was quantified using Cellsens software (Olympus, Shinjuku City, Tokyo, Japan), to quantify the distance of the alveolar bone in direct contact with the implant in addition to the total length of the implant at the bone level. BIC% was calculated by determining the fraction of bone contact relative to the total implant length. BIC% data were compared between different groups for statistical significance and the results were presented as mean ± standard deviation.

### Statistical analysis

Chi-square analysis was conducted to analyze the results from clinical evaluation to describe the relationship between the presence and absence of a clinical parameter for all the groups. Statistical analysis of distance between implant head and bone crest, tooth separation, BV/TV%, and histomorphometry was accomplished by one-way analysis of variance with a Post hoc Tukey test to compare different groups if samples were normally distributed by Shapiro–Wilk Normality test. If the samples did not follow a normal distribution, non-parametric Kruskal–Wallis test was performed with Dunn’s multiple comparisons test. Tukey/Dunn tests made comparisons to evaluate significance between groups. All statistical tests were run on GraphPad Prism 9.0 software (GraphPad Software Inc., San Diego, CA, USA) with a confidence level (α) of 0.05. p values were used to determine significance between groups.

### Ethical statement

Animal surgeries including pre- and post-operative care were conducted under the guidance and authorization of Institutional Animal Care and Use of Laboratory Animals (IACUC #20-09) according to NIH Guide for the Care and Use of Laboratory Animals. All the experiments were approved by ethics committee and the study also follows the ARRIVE guidelines as required for all in vivo work.

## Data Availability

Data available within the article. Raw data that support the findings of this study are available from the corresponding authors DCR and BL upon reasonable request.
